# Arabic digits and spoken number words: Timing modulates the cross-modal numerical distance effect

**DOI:** 10.1177/1747021819854444

**Published:** 2019-06-15

**Authors:** Chia-Yuan Lin, Silke M Göbel

**Affiliations:** 1 Department of Psychology, University of York, York, UK; 2 Department of English Language and Linguistics, University of Kent, Canterbury, UK

**Keywords:** Numerical distance effect, cross-format correspondence, spoken number words, same–different task, stimulus onset asynchrony, physical similarity

## Abstract

Moving seamlessly between spoken number words and Arabic digits is common in everyday life. In this study, we systematically investigated the correspondence between auditory number words and visual Arabic digits in adults. Auditory number words and visual Arabic digits were presented concurrently or sequentially and participants had to indicate whether they described the same quantity. We manipulated the stimulus onset asynchronies (SOAs) between the two stimuli (Experiment 1: −500 ms to +500 ms; Experiment 2: −200 ms to +200 ms). In both experiments, we found a significant cross-modal distance effect. This effect was strongest for simultaneous stimulus presentation and decreased with increasing SOAs. Numerical distance emerged as the most consistent significant predictor overall, in particular for simultaneous presentation. However, physical similarity between the stimuli was often a significant predictor of response times in addition to numerical distance, and at longer SOAs, physical similarity between the stimuli was the only significant predictor. This shows that SOA modulates the extent to which participants access quantity representations. Our results thus support the idea that a semantic quantity representation of auditory and visual numerical symbols is activated when participants perform a concurrent matching task, while at longer SOAs participants are more likely to rely on physical similarity between the stimuli. We also investigated whether individual differences in the efficiency of the cross-modal processing were related to differences in mathematical performance. Our results are inconclusive about whether the efficiency of cross-format numerical correspondence is related to mathematical competence in adults.

## Introduction

In everyday life, we easily and effortlessly switch between different number formats, for example, between a spoken number word (e.g., “three”) and an Arabic digit (e.g., 3). It has been suggested that frequent pairings of particular auditory and visual symbols might lead to an integrated, bimodal audiovisual percept (Holloway & Ansari, 2015). This has also been proposed for the pairing of letters and their corresponding sounds. Evidence for the automatic audiovisual integration between visually presented letters and auditorily presented letter–sounds comes from neuroimaging and electroencephalography (EEG) studies (Froyen, van Atteveldt, Bonte, & Blomert, 2008; van Atteveldt, Formisano, Blomert, & Goebel, 2007). The timing and efficiency of this letter–sound integration have been found to be related to reading development and variations in reading ability (for a review, see Blomert, 2011). Just like for the acquisition of letter–sound pairings, young children also take time to learn the correspondence between number words and Arabic digits before they become fast and efficient (Knudsen, Fischer, Henning, & Aschersleben, 2015). Thus, it is not unreasonable to expect a relationship between the ease with which individuals move between Arabic digits and spoken number words and their mathematical ability.

This is exactly what Sasanguie and Reynvoet (2014) found. They tested adults’ performance on a simultaneous digit–number word matching task and found that the better the adults’ mathematical performance, the faster they performed the matching task between visual Arabic digits and spoken number words. They also assessed adults’ efficiency in matching pairs of dots and number words and pairs of letters with speech sounds. Performance on those tasks was not related to participants’ mathematical performance. This indicates that the efficiency of accessing the correspondence between spoken number words and Arabic digits, but not the correspondence between dots and number words, is related to mathematical performance in adults.

In contrast to letter–speech sound matching, Arabic digits and spoken number words carry meaning. A large body of research suggests that when participants process numerical stimuli, they automatically activate their numerical size, that is, the numerical magnitude (for a review, see Tzelgov & Ganor-Stern, 2005). One influential idea of how numerical symbols acquire their meaning is that symbolic numbers are mapped onto non-symbolic internal representations of magnitude and that those internal magnitudes are represented as imprecise distributions on a mental number line (Dehaene, 1992; Lyons, Ansari, & Beilock, 2012). This proposal can account for a robust effect reliably found during number comparison, the distance effect (Moyer & Landauer, 1967): reaction times are typically longer and accuracies are lower for numerical judgements when two numbers are numerically closer than when they are further away from each other (e.g., Dehaene & Akhavein, 1995; van Opstal & Verguts, 2011).

In the mental number line model, when two symbolic numbers are numerically closer, their internal distributions are more likely to overlap on the mental number line and this is supposed to make it harder to differentiate between them and thus lead to lower accuracies and longer reaction times, that is, a larger distance effect. The numerical distance effect has also been observed for mixed number formats, for example, between Arabic digits and written number words (e.g., Dehaene & Akhavein, 1995). This has been taken as evidence that an abstract representation of numerical magnitude is automatically accessed to perform cross-format number comparison (e.g., Tzelgov & Ganor-Stern, 2005).

The existence of an abstract numerical representation and whether this representation is automatically activated when individuals process numbers, however, has been the focus of considerable debate (see, for example, Cohen Kadosh & Walsh, 2009). Classical models of the mental architecture for number processing differ on this point. Some models assume that all numerical inputs must first be transcoded into an abstract, and amodal magnitude representation before number comparison can take place (e.g., McCloskey, 1992). Thus, these models predict a numerical distance effect in all numerical tasks. In contrast, other models assume multiple, specific representations for different numerical inputs (e.g., Campbell, 1994; Campbell & Clark, 1988; Campbell & Epp, 2004; Cohen, Warren, & Blanc-Goldhammer, 2013) with direct connections between different representations without the need to pass through an abstract magnitude representation. A numerical distance effect is not always expected.

The majority consensus in the literature used to be that there was strong empirical evidence for the existence of abstract magnitude representation, for example, from cross-format distance effects. However, the nature of the evidence has been seriously questioned over the last decade. Cohen Kadosh and Walsh (2009), for example, revisited the interpretation of several classic papers on cross-format distance effects showing that the evidence might have been less clear than expected. For example, in the classic study by Dehaene and Akhavein (1995), participants were asked to perform same–different judgements on pairs of numerals (Arabic digits and written number words, for example, 2–TWO). There was indeed a significant distance effect in both pure (e.g., 2–4, TWO–FOUR) and mixed conditions (e.g., 2–FOUR), when participants were asked to perform numerical matching. However, there was no significant distance effect on mixed trials when participants were asked to perform a physical matching task, which is the crucial condition when one postulates an abstract magnitude representation that is automatically activated. Similarly, Ganor-Stern and Tzelgov (2008) found no significant distance effect when participants had to perform a physical same–different task on pairs of Arabic and Indian numerical symbols.

More recently, evidence has also been accumulating showing that magnitude representations might not always be automatically activated even in numerical tasks (Cohen, 2009; Defever, Sasanguie, Vandewaetere, & Reynvoet, 2012; García-Orza, Perea, Mallouh, & Carreiras, 2012; Wong & Szűcs, 2013). For example, Sasanguie and Reynvoet (2014) used a cross-format same–different task and failed to find a significant numerical distance effect in the digit–number word matching condition. In their study, reaction times were not significantly longer for comparing a spoken number word and an Arabic digit when they were numerically closer to each other than when they were further away from each other in quantity. They interpreted the absence of a significant distance effect as evidence that the correspondence between spoken number words and Arabic digits in adults is so highly overlearned, fast, and automatic, that it is unnecessary to access the shared magnitude representation for same–different judgements.

Furthermore, Cohen (2009) contributed to the debate by showing that even the presence of a distance effect by itself is not sufficient evidence that an abstract magnitude representation has been accessed. In two numerical same–different experiments with single Arabic digits, participants’ response time (RT) data were better predicted by the physical similarity between the target and distractor number than by their numerical distance. Cohen showed that the physical similarity and the numerical distance of single digits is highly correlated. Thus, the presence of a numerical distance effect, as reported in many previous studies, does on its own not necessarily indicate that number magnitude has been accessed. Cohen’s study highlights the need to investigate whether a numerical distance effect is still present once physical similarity between digits has been controlled for. Cohen et al. (2013) extended the physical similarity argument to cross-format number comparison and proposed how participants might solve cross-format same–different tasks without accessing semantic magnitudes. Participants, upon hearing a spoken number word, might convert the spoken number word into the corresponding Arabic digit and then directly compare this digit representation with the visually presented Arabic digits based on the physical overlap between them (physical similarity). This predicts a correlation of RTs with the visual similarity between both numbers (*P_visual_*). Alternatively, participants might convert the presented Arabic digit into a spoken number word and compare this with the actual presented spoken number word. In that case, RTs would be predicted by the auditory similarity between the two number words (*P_auditory_*). In both situations, no access to the semantic magnitude representation is necessary.

Cohen et al. (2013) tested their prediction with a cross-format same–different task with single-digit numbers. First, they presented participants with a spoken number word. Then, 500 ms after the onset of the spoken number word, participants saw an Arabic digit presented on the screen and had to perform a same–different number judgement. In this experiment, only the physical similarity between the Arabic digits was a significant predictor of participants’ RTs. These findings provide strong evidence that participants converted the spoken number words into Arabic digits and that they then compared the two Arabic digits directly based on their physical similarity. This study suggests that even cross-format same–different tasks can be solved without accessing the semantic magnitude.

Following Cohen et al.’s (2013) argument, participants might be expected to use different strategies depending on which format is presented first to them. Cohen et al. (2013) only tested the condition in which auditory number words were presented first. In this situation, converting the number words into the corresponding Arabic digit, that is, into the format in which the second stimulus was always presented, is an efficient strategy. However, do participants change their strategy when the order of the two stimuli is reversed? If the first stimulus is an Arabic digit and the second stimulus a spoken number word, are they then converting the Arabic digit into the corresponding number word? In that case, we predict the auditory similarity between the two number words rather than the visual similarity between the Arabic digits to be a significant predictor of RTs.

In this study, we varied the order of spoken number words and Arabic digits and investigated whether RTs are best predicted by numerical distance, visual or auditory similarity between the stimuli separately for each cross-format condition (i.e., Arabic digit–spoken number word and spoken number word–Arabic digit).

Furthermore, a common way to examine the relationship between multi-sensory inputs is to manipulate the time interval between the sequential presentation of stimuli. It has been shown that the temporal proximity between stimuli influences behavioural responses when there is a well-learned correspondence between multi-sensory inputs (e.g., ten Oever, Sack, Wheat, Bien, & van Atteveldt, 2013; van Wassenhove, Grant, & Poeppel, 2007). For example, a fusion perception of video clips of lip movements and speech sounds, that is, cross-modal integration, only happens when bimodal inputs are displayed within a short time interval (e.g., within less than 250 ms) from each other, and the congruency of lip movements and speech sounds modulates the simultaneity judgement (van Wassenhove et al., 2007). These findings suggest a limited time window between two sequentially presented stimuli, outside of which cross-modal integration might not happen automatically anymore. To the best of our knowledge, currently only two studies have investigated cross-format number processing with spoken number words and Arabic digits. Sasanguie and Reynvoet (2014) did not manipulate the time interval between the auditory number word and Arabic digit. In their study, spoken number words and Arabic digits were always presented simultaneously. Cohen et al. (2013) always presented the Arabic digits 500 ms after the onset of the auditory number word. While Sasanguie and Reynvoet (2014) found no significant distance effect with simultaneous presentation, Cohen et al. (2013) found a significant distance effect with a stimulus onset asynchrony (SOA) of 500 ms. However, in their study, the distance effect was entirely explained by the physical similarity between the stimuli and not by their numerical distance. It is conceivable that participants use both perceptual similarity and access to an abstract magnitude representation in parallel but that these operate on different time scales (e.g., García-Orza et al., 2012).

The aim of this study was to systematically manipulate the length of the time window between spoken number words and Arabic digits and, in particular, to include SOAs between 0 and 500 ms. This design allows us to investigate the temporal dynamics of the cross-modal number matching task and thus may solve some of the inconsistencies found in the literature between studies that employed simultaneous presentations (e.g., Sasanguie & Reynvoet, 2014) and those that used sequential presentations (e.g., Cohen et al., 2013).

Thus, we conducted two same–different experiments with different ranges of SOA. In the first experiment, we manipulated the SOA from −500 ms (a visual digit was displayed first) to 0 ms (two stimuli are displayed simultaneously) to 500 ms (an auditory number word was displayed first). In the second experiment, we used a shorter temporal interval between bimodal numerals, from −200 ms to 200 ms with higher temporal resolution. In addition, we were interested in whether we would observe a cross-modal distance effect. If the judgement for the audiovisual matching task is made without magnitude processing, as suggested by Sasanguie and Reynvoet (2014), then we should observe no distance effect in any of the SOA conditions.

In contrast, if we observe a distance effect, then we need to test whether this reaction time pattern is predicted better by the numerical distance between the stimuli or rather by the physical similarity between the stimuli. We directly compare those alternative explanations by following the procedures described in Cohen et al. (2013).

In addition, we included a standardised mathematical test and, based on findings by Sasanguie and Reynvoet (2014), expected that participants with better mathematical performance would respond faster in the audiovisual matching task.

## Experiment 1

### Method

#### Participants

Forty-three native English-speaking university students (25 females; age range: 18-36 years; mean age = 20.86 years, *SD* = 3.04 years) participated for either monetary compensation (£6) or course credit. All participants gave written informed consent. Both experiments received ethical approval from the Ethics Committee, Department of Psychology at the University of York, UK.

#### Stimuli and procedure

Stimuli presentation and data recording were controlled by Presentation^®^ (Version 17.2; www.neurobs.com). The numbers 2, 3, 4, 5, 6, and 8 were used in visual and auditory format (7 was excluded because the auditory number word contains two syllables). The visual Arabic digit was displayed in the middle of an 18.3-in screen, in white against a black background, in Arial font, 48 pt. The sound for each auditory number word was recorded digitally by a male native English speaker in a soundproof booth. The sound files were manipulated by using Cool Edit 2000 to be roughly equal in length (around 450 ms). The sounds were played binaurally through a headphone.

**Figure 1.**
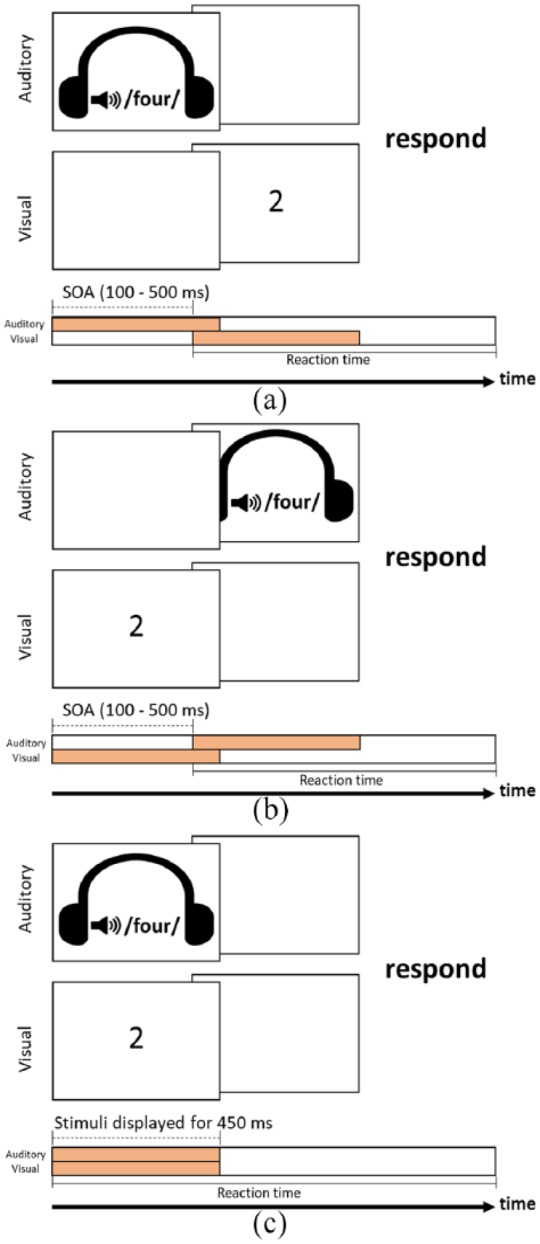
Experimental procedure for the single digit–spoken number word matching task: (a) an example for unmatched trials with an auditory number word displayed first (AV condition), (b) an example with a visual Arabic digit displayed first (VA condition), and (c) an example in the condition where the auditory and the visual stimulus are displayed simultaneously.

On each trial, a fixation cross (white, 48 pt) was displayed first in the centre of the screen. After 500 ms, the fixation disappeared and bimodal numerals were displayed. An Arabic single digit was presented at the centre of the screen for 450 ms and a spoken number word was played for around 450 ms (mean length of sounds = 449.43 ms, *SD* = 2.64 ms, range from 444 to 459 ms). Nine different SOAs were used: −500, −300, −200, −100, 0, +100, +200, +300, and +500 ms. These manipulations of SOAs led to three types of sequences: visual-first-then-auditory (VA) condition with negative SOAs (−500, −300, −200, and −100 ms), the auditory-first-then-visual (AV) with positive SOAs (+100, +200, +300, and +500 ms), and simultaneous display (0 ms; see Figure 1). The inter-trial interval was 500 ms.

Every combination of each digit for each SOA condition was displayed in a random order across blocks (9 blocks in total, 60 trials for each block). The sequence of stimulus presentation was randomly generated but fixed across participants. The experiment started with a 12-trial practice block. Using a standard QWERTY keyboard, participants were instructed to respond by key buttons pressing (“Z” and “/”) “same” when they saw and heard the same number (matching trials) and to respond “different” when the written digit and the sound of number word were different (non-matching trials). The button allocation was counter-balanced between subjects. To balance the same and different responses, the “same” pair (e.g., trials when they saw a digit “2” and heard a number word “two”) was displayed five times in each SOA, whereas there was only one trial for each “different” pair (e.g., 2–three, 2–four, 2–five, 2–six, and 2–eight). In total, there were 540 trials. Note that the reaction time was measured as the time between the onset of the second stimulus and the first response.

Individual math performance was assessed with the Math Computation subtest of the Wide Range Achievement Test (WRAT-4, Wilkinson & Robertson, 2006) after the computerised task. Participants were asked to solve as many arithmetic questions as possible in 15 min (a maximum of 40 questions). Calculators were prohibited. The age-standardised score was calculated for each participant following the test manual. The delayed and immediate test–retest reliability reported in the test manual is *r* = .83 and .88, respectively.

#### Data analyses

In addition to standard analyses of variance (ANOVAs) on participants’ RTs, we performed follow-up model fitting analyses to compare whether the RT pattern observed was best predicted by numerical distance or physical similarity. We used two perceptual (*P*) predictors and one numerical (*N*) predictor: $P_{digit}$, $P_{auditory}$, and $N_{D}$. For $P_{digit}$, we used the values reported in Cohen (2009). These similarity values are based on the number of overlapping lines when comparing Arabic digits in an old digit clock font: $P_{digit}=O/C$. *O* represents the number of overlapping lines and *C* represents the number of non-overlapping lines. Thus, a larger $P_{digit}$ means the Arabic digits are more similar. $P_{auditory}$ is a function accounting for auditory similarities among different auditory number words and is based on the values reported in Cohen et al. (2013). It represents the distance between different spoken number words, thus a larger value means two auditory words are less similar. $N_{D}$ accounts for a linear numerical difference between numbers $(N_{D}=larger\;number-smaller\;number).$ We chose a linear rather than logarithmic (e.g., Welford function) measure here for two reasons. First, the calculation procedure of the linear numerical distance is more similar to the procedure used to calculate the physical similarities for the perceptual predictors described above. Second, it has been shown that a model with an equal-spaced, that is, linear representation fits the data better than a logarithmic model (Cohen & Quinlan, 2016). As described above, we used 15 number pairs in both experiments. The Pearson correlation coefficients between $P_{digit}$ and $P_{auditory}$ of these number pairs was .01, between $N_{D}$ and *P_digit_* was −.05, and between *N_D_* and $P_{auditory}$ was −.62.

We closely followed the analysis procedure described in Cohen et al. (2013). We conducted a simultaneous mixed model analysis (*lmer* function in R; Bates, Mächler, Bolker, & Walker, 2015), using three different functions ($P_{digit}$, $P_{auditory}$, and $N_{D}$) as predictors with RT of each trial as dependent variable and treating subject as a random effect variable. We logarithmically transformed the RTs and normalised all four predictors separately. To further address the influence of the asynchrony between audiovisual numerals in the current matching task, reaction times of VA, AV, and 0 SOA (when numerals were presented simultaneously) were used as dependent variable in separate linear mixed effect regression analyses. The significance was estimated by using the *lmerTest* package in R (Kuznetsova, Brockhoff, & Christensen, 2017). The family-wise error rate was controlled by Bonferroni correction, according to the number of fixed factors (Cohen et al., 2013). The marginal *R*^2^ values (i.e., the variance explained by fixed factors) are also reported (*r.squaredGLMM* function in R; Nakagawa & Schielzeth, 2013).

### Results

#### Reaction time

Two participants were excluded because they missed a large number of trials (48.9% and 14.6%, respectively), and three were dropped due to the experimental programme crashing. Trials with RTs smaller than 250 ms and larger than 1,500 ms were excluded from further analyses (1.65% of total trials). Few errors were made (mean accuracy: 96.6%, *SD* = 2.3%). A significant lower accuracy rate was found for matching trials than non-matching trials (95.3% vs 97.7%), *t*(37) = −5.23, *p* < .001, *d* = −0.85 (see Table 1).

**Table 1.** Means and standard deviations of accuracy rates and reaction times by SOA.

	Experiment 1 (*N* = 38)								
	VA					AV			
SOA (ms)	500	300	200	100	0	100	200	300	500
Accuracy	.98 (.03)	.97 (.03)	.96 (.03)	.96 (.03)	.96 (.04)	.95 (.04)	.97 (.03)	.96 (.03)	.97 (.03)
RT (ms)	602 (103)	604 (107)	612 (109)	633 (108)	680 (118)	619 (111)	559 (110)	525 (97)	491 (102)
	Experiment 2 (*N* = 49)								
	VA					AV			
SOA (ms)	200	150	100	50	0	50	100	150	200
Accuracy	.95 (.03)	.96 (.03)	.95 (.04)	.94 (.05)	.94 (.03)	.95 (.04)	.95 (.04)	.95 (.04)	.94 (.05)
RT (ms)	625 (81)	633 (77)	644 (86)	658 (83)	677 (82)	646 (81)	603 (83)	577 (82)	551 (79)

SOA: stimulus onset asynchronies; VA: visual first-then-auditory condition; AV: auditory first-then-visual condition; RT: response time.

A 2 (number matching: same and different) by 9 (SOAs: −500, −300, −200, −100, 0, +100, +200, +300, and +500 ms) repeated-measures ANOVA was conducted for correct RTs (*M* = 591 ms, *SD* = 105 ms). The significant main effect of matching, *F*(1, 37) = 86.86, *p* < .001, $\eta_{p}^{2}=.70$, showed a higher mean RT for different trials (*M* = 618 ms, *SD* = 110 ms) than for same trials (*M* = 564 ms, *SD* = 102 ms). The main effect of SOAs was also significant, *F*(8, 296) = 194.89, *p* < .001, $\eta_{p}^{2}=.84$. The RTs were longer the closer the SOA was to 0 ms and was the longest at 0 ms SOA. The post hoc comparisons between mean RTs of adjacent SOA conditions showed that only SOA conditions −500 ms and −300 ms and −300 ms and −200 ms were not significantly different from each other, all other comparisons between mean RTs for adjacent SOA conditions were significant (all *ps* < .05). The interaction effect between matching and SOAs was not significant, *F*(8, 296) = 1.79, *p* = .08, $\eta_{p}^{2}=.05$. All post hoc comparisons were Bonferroni corrected.^1^

To examine the modality effect of stimulus order, a 2 (matching: same vs different) by 2 (modality order: VA vs AV) by 4 (SOA: 100, 200, 300, and 500 ms) ANOVA was conducted. The concurrent display (i.e., SOA = 0 ms) was excluded in this analysis. The results showed, as in the previous ANOVA, a significant main effect of matching, *F*(1, 37) = 92.03, *p* < .001, $\eta_{p}^{2}=.71$. There was a significant linear trend of SOA, *F*(1, 37) = 316.20, *p* < .001, $\eta_{p}^{2}=.90$, indicating that RTs changed with SOA. The interaction between modality order and SOA was also significant, *F*(3, 111) = 77.26, *p* < .001, $\eta_{p}^{2}=.68$. Further post hoc analyses showed that the interaction was due to different “decreasing rates” depending on modality order when SOA increased:^2^ The RTs of AV conditions dropped quickly when SOA increased, whereas RTs of VA conditions had a somewhat similar decreasing trend but the decline was less steep.

#### Classical analyses of the distance effect

**Figure 2.**
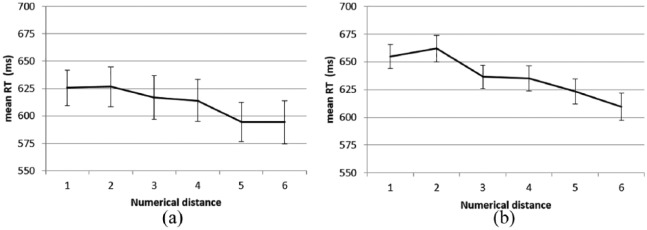
The cross-modal numerical distance effect in the single digit–number word matching task in (a) Experiment 1 and (b) Experiment 2. The error bars indicate ±1 *SE*.

Only different trials were included into the data analysis for the distance effect. A one-way repeated-measures ANOVA of RTs by numerical distance (1-6) revealed a significant main effect of distance, *F*(5, 185) = 9.45, *p* < .001, $\eta_{p}^{2}=.81$. The post hoc analyses showed that except for distance 1 and 2, distance 3 and 4, and distance 5 and 6, mean RTs differed significantly between all adjacent distances (all *ps* < .05). The linear trend was significant, *F*(1, 37) = 34.14, *p* < .001, *$\eta_{p}^{2}=.48$*. These results showed that the larger the numerical distance between the Arabic digit and the auditory number word, the shorter the RTs (see Figure 2a), indicating the presence of a classic distance effect.

**Figure 3.**
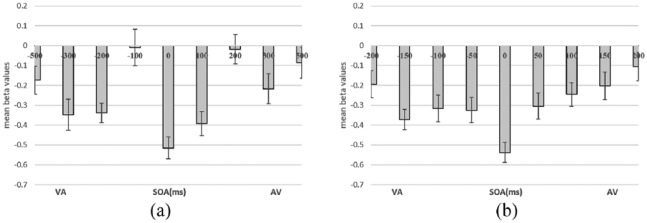
The mean beta values of the cross-modal numerical distance effect by SOA for (a) Experiment 1 and (b) Experiment 2. The error bars indicate ±1 *SE*. VA indicates that the visual Arabic digit was displayed first, whereas AV indicates that the auditory number word was displayed before the visual digit.

To further investigate the distance effect for each participant in different SOA conditions, we calculated the distance effect separately for each participant in each SOA condition. First, as for the ANOVA described above, the RT data were divided by SOA and numerical distance for each subject. Then, for each subject in each SOA condition separately, a beta value was calculated using a linear regression predicting their overall mean RTs based on their RTs for each numerical distance. Compared to using averaged RT across subjects for the regression model, this approach considers individual differences in the distance effect in each SOA condition (Lorch & Myers, 1990). A negative beta value is an indicator of a classic distance effect, that is, larger RTs with smaller distances. Note that the more negative the beta value, the stronger the distance effect. Then, we calculated the mean beta value for each SOA condition by averaging the beta values across participants (see Figure 3a).

A one-sample *t*-test showed that the mean beta values averaged over all SOA conditions (mean beta values = −.48, *SE* = .05, 95% confidence interval = [−.62, −.34]) was significantly different from zero, *t*(37) = −6.87, *p* < .001, *d* = −1.11. This indicated that the numerical distance effect was significant when aggregating the RTs of all SOA conditions. One-sample *t*-tests by SOA showed that the distance effect was significantly different from zero in all SOAs (*p*s < .007, *d* < −0.46, Holm–Bonferroni corrected for multiple comparisons) except for −500 (*p* = .019), −100 ms (*p* = .92), +200 ms (*p* = .80), and +500 ms SOAs (*p* = .28).

To compare the beta values between the different SOA conditions, a one-way ANOVA of beta values by SOAs (9 levels; from −500 to +500 ms) was conducted. The results showed a significant main effect, *F*(8, 296) = 6.34, *p* < .001, $\eta_{p}^{2}=.15$. The data pattern indicated a more negative beta value, that is, a stronger distance effect, when the auditory and visual stimuli were displayed simultaneously, and then became more positive, that is, a weaker distance effect, when the SOA increased. The post hoc analyses showed that the beta value of 0 ms SOA was significantly more negative than others (all *ps* < .05) except for −300 ms (*p* = .062) and +100 ms SOA (*p* = .12).

To further investigate the possible influence of the order of the stimulus modality on the distance effect, a 2 (modality order: VA and AV) by 4 (SOA: 100, 200, 300, and 500 ms) ANOVA was conducted. A significant interaction between the order of modality and the SOA was found, *F*(3, 111) = 7.81, *p* < .001, $\eta_{p}^{2}=.17$. Post hoc analyses showed that the interaction emerged because of opposite patterns in the distance effect depending on order of modality for 100 and 200 ms SOA: the distance effect in the VA condition was significantly more negative than in AV condition for 100 ms SOA (*p* < .01), whereas distance effect in the VA condition was significantly larger than in the AV condition for 200 ms SOA (*p* < .01). There was no significant main effect of order of modality, *F*(1, 37) = 0.48, *p* = .49, $\eta_{p}^{2}=.01$, or SOA, *F*(3, 111) = 1.88, *p* = .14, $\eta_{p}^{2}=.05.$ There was also no significant linear trend of SOA, *F*(1, 37) = 0.27, *p* = .61, $\eta_{p}^{2}=.01$.

#### Model fitting

When visual numerals were presented earlier than auditory numerals (VA), the slope for $N_{D}$ (slope = −0.007, *t* = −4.03, *p* < .001), $P_{digit}$ (slope = −0.005, *t* = −3.24, *p* = .0012), and the intercept (2.789, *t* = 243.84, *p* < .001) were significant (marginal *R*^2^ = .005; see Table 2). When auditory numerals were presented first (AV), the slope for $N_{D}$ (slope = −0.008, *t* = −4.13, *p* < .001) and the intercept (2.736, *t* = 223.93, *p* < .001) were significant (marginal *R*^2^ = .003). When audiovisual numerals were presented simultaneously (0 SOA), the slope for $N_{D}$ (slope = −0.016, *t* = −4.70, *p* < .001), $P_{digit}$ (slope = −0.007, *t* = −2.46, *p* = .014), and the intercept (2.829, *t* = 248.44, *p* < .001) were significant (marginal *R*^2^ = .035). To compare the current results with previous findings from Cohen et al. (2013), we also examined different models at AV500, which was the condition used by Cohen and colleagues. We decided to also examine VA500 because this condition should show the largest difference to the AV500 condition if participants relied on different strategies for their numerical judgements with the change of SOA. For the VA500 condition, the slope for $P_{digit}$ (slope = −0.007, *t* = −2.24, *p* = .025) was marginally significant^3^ and the intercept (2.781, *t* = 225.35, *p* < .001) was significant (marginal *R*^2^ = .004). For the AV500 condition, only the intercept (2.688, *t* = 211.23, *p* < .001) was significant (marginal *R*^2^ = .001).

**Table 2.** The slopes and *t*-values for the linear mixed modelling results in Experiment 1 and Experiment 2.

Experiment 1						
	VA			AV		
	Slope	*t*		Slope	*t*	
*N_D_*	−0.007	−4.03**		−0.008	−4.13**	
*P_digit_*	−0.005	−3.24*		−0.003	−2.08	
*P_auditory_*	−0.001	−0.65		−0.003	−1.43	
	–500		0		+500	
	Slope	*t*	Slope	*t*	Slope	*t*
*N_D_*	−0.002	−0.62	−0.016	−4.70**	−0.003	−0.70
*P_digit_*	−0.007	−2.24	−0.007	−2.46*	0.002	0.69
*P_auditory_*	0.002	0.63	0.006	1.82	−0.001	−0.34
Experiment 2						
	VA			AV		
	Slope	*t*		Slope	*t*	
*N_D_*	−0.010	−6.77**		−0.007	−4.19**	
*P_digit_*	−0.003	−2.64*		−0.002	−1.84	
*P_auditory_*	<–0.001	−0.25		0.002	1.11	
	–200		0		+200	
	Slope	*t*	Slope	*t*	Slope	*t*
*N_D_*	−0.003	−1.13	−0.021	−7.52**	0.003	0.88
*P_digit_*	−0.007	−3.23*	−0.005	−2.32	0.001	0.32
*P_auditory_*	0.003	1.09	−0.001	−0.39	0.011	3.70**

VA: visual first-then-auditory condition; AV: auditory first-then-visual condition.

“−” represents VA conditions, while “+” represents AV conditions. *N_D_* represents the linear distance between numbers. *P_digit_* represents the physical similarity between Arabic digits. *P_auditory_* represents the physical similarity between auditory number words.

* *p *⩽ .0166 (Bonferroni corrected for three predictors); **p ⩽ .001.

#### Relationship to mathematical performance

**Figure 4.**
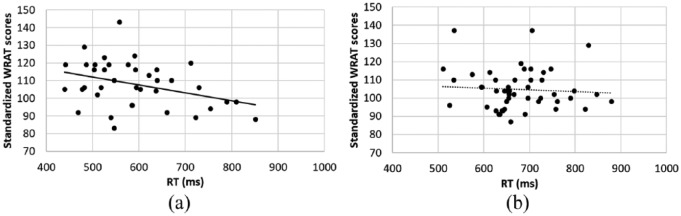
The scatter plots for the standardised WRAT scores and the overall mean RTs of the digit–number word matching task. (a) The solid line indicates the significant correlation (*r* = −.36, *p* = .028) between the WRAT scores and the RTs of the digit–number word matching task in Experiment 1. (b) There was no significant correlation between the WRAT scores and the overall mean RTs of the matching task in Experiment 2 (*r* = −.04, *p* = .77).

To investigate the relationship between the mathematical competence and the bimodal numerical matching task, a Pearson correlation analysis was conducted for the standardised score of the WRAT-4 math computation subtest (mean WRAT standardised score: 107.92, *SD* = 12.97, range from 83 to 143) and the RT of the bimodal matching task. The WRAT scores were negatively correlated with the overall correct RT for the digit–number word matching task, *r*(38) = −.36, *p* = .028 (see Figure 4a). The WRAT scores were also negatively correlated with overall beta values (*r*(38) = −.41, *p* = .01), that is, a larger distance effect over all SOAs was associated with better performance on the math computation test. However, there was no significant relationship between the size of the distance effect (beta values) and math performance when we only analysed the trials with simultaneous presentation (SOA = 0; *r*(38) = .03, *p* = .84).

## Experiment 2

In the second experiment, we aimed to further examine shorter SOAs, to have a better temporal resolution for observing the modulation of the distance effect (van Wassenhove et al., 2007). Also, while we found a significant correlation between mathematical performance and participants’ variations in speed in the matching task in Experiment 1, this relationship might have been due to individual differences in other factors affecting performance on the matching task such as non-verbal IQ and general speed of processing, which we did not measure in the first experiment. Hence, in the second experiment, we included a non-verbal IQ test and a general processing speed task. The aim was to investigate the relationship between mathematical performance and performance on the matching task while controlling for these potential confounding variables.

### Method

#### Participants

Fifty-one native English-speaking university students of the University of York (41 females; age range: 18-30 years; mean age = 19.84 years, *SD* = 1.83 years) participated for either monetary compensation (£6) or 1 hr course credit.

#### Stimuli and procedure

The same procedure as in Experiment 1 was used. However, stimuli presentation and data recording were now controlled by MATLAB with Psychophysics Toolbox extensions (Matlab Psychtoolbox-3; www.psychtoolbox.org), and importantly, we used different SOA conditions: nine shorter SOAs were used in the present experiment, they were −200 ms, −150 ms, −100 ms, −50 ms, 0 ms, +50 ms, +100 ms, +150 ms, and +200 ms.

In addition to the WRAT-4 math computation test, a task measuring general processing speed task and non-verbal IQ were added. During the processing speed task, participants were instructed to press the space bar as fast as possible as soon as they saw a white square displayed on a black, 18.3-in screen. The square was 50 × 50 pixels in size and was presented in the centre of the screen. The square disappeared as soon as the participant responded and was followed by a blank screen with an inter-stimulus interval (ISI) ranging from 500 to 1,500 ms (mean ISI = 1,000 ms). A 5-trial practice block was given before the 20-trial main block, the mean RT was measured as an indicator of processing speed. The task took about 1 min to complete. For measuring non-verbal IQ, we used the matrix reasoning subtest of the Wechsler Abbreviated Scales of Intelligence-II (WASI-II; Wechsler, 2011). A series of shapes with one missing part was displayed to participants. Participants had to choose a(n) shape/element that completed the pattern of the shapes they saw. The experimenter tested each participant by following the test manual. There was no time limit for this test. The maximum raw score is 28 if all questions are answered correctly. The raw scores were then transferred to a standardised *T-*scores based on age norms. The split-half and test–retest reliability reported in the test manual is *r* = .90 and .75 for adults, respectively.

### Results

#### Reaction time

Two participants were excluded due to low accuracy (lower than 3 standard deviations of the mean). RTs smaller than 250 ms and larger than 1,500 ms were excluded in further analyses (0.7% of total trials). Few errors were made (mean accuracy: 94.8%, *SD* = 3.0%). A significant lower accuracy rate was found for matching than non-matching trials (94.4% vs 96.5%), *t*(48) = −5.96, *p* < .001, *d* = −0.85 (see Table 1).

A 2 (matching: same and different) by 9 (SOAs: −200, −150, −100, −50, 0, +50, +100, +150, and +200 ms) repeated-measures ANOVA was conducted for correct mean RTs (*M* = 624 ms, *SD* = 80 ms). The significant main effect of matching (*F*(1, 48) = 84.71, *p* < .001, $\eta_{p}^{2}=.64$) showed higher RTs for different (*M* = 646 ms, *SD* = 76 ms) than for same trials (*M* = 602 ms, *SD* = 86 ms). The main effect of SOAs was also significant (*F*(8, 384) = 207.24, *p* < .001, $\eta_{p}^{2}=.81$). The RTs were longer the closer the SOA was to 0 ms SOA. The post hoc analyses showed that the comparisons between an SOA and the one next to itself were all significant (all *ps* < .05), that is, the RT of concurrent displays was the longest, and then, RTs decreased with the increase in SOA. The interaction effect between number matching and SOAs was also significant (*F*(8, 384) = 2.50, *p* = .012, $\eta_{p}^{2}=.05$).^4^

To examine the modality effect of stimulus order, a 2 (matching: same vs different) by 2 (modality order: VA vs AV) by 4 (SOA: 50, 100, 150, and 200 ms) ANOVA was conducted. The concurrent display (i.e., 0 ms SOA) was excluded in this analysis. There was a significant linear trend of SOA (*F*(1, 48) = 659.82, *p* < .001, $\eta_{p}^{2}=.93$), showing that RTs decreased linearly with SOA. A significant three-way interaction was found between matching, modality order, and SOA (*F*(3, 144) = 4.29, *p* = .006, $\eta_{p}^{2}=.08$). Similar to what we found in the first experiment, RTs of AV conditions decreased significantly when SOA increased, whereas RTs of VA conditions had a similar decreasing trend but the declining rate was milder.^5^

#### Classical analyses of the distance effect

A one-way ANOVA of RTs by different numerical distances revealed a significant main effect of distance (*F*(5, 240) = 32.57, *p* < .001, $\eta_{p}^{2}=.40$). The post hoc analyses showed that except for distance 1 and 2 (*p* = .11) and distance 3 and 4 (*p* = .74), all other comparisons were significant (all *ps* < .05). The linear trend of distance was significant too (*F*(1, 48) = 111.86, *p* < .001, $\eta_{p}^{2}=.70$). These results showed that the larger the numerical distance between the auditory number word and the visual Arabic digit, the shorter the RTs, indicating a classic distance effect (see Figure 2b).

The beta values for the numerical distance by SOA were calculated following the procedure described in Experiment 1 separately for each individual (mean beta value = −.64, *SD* = .31, 95% confidence interval = [−.73, −.56]; see Figure 3b). The mean beta value of all conditions was significantly different from zero (*t*(48) = −14.58, *p* < .001, *d* = −2.08), showing the numerical distance effect when aggregating the RTs of all SOA conditions. One-sample *t*-tests by SOA showed that the distance effect was significantly different from zero in all SOAs (*p*s < .007, *d* < −0.41, Holm–Bonferroni corrected for multiple comparisons) except for +200 ms (*p* = .15).

To compare the beta values of SOA conditions, a one-way ANOVA of beta values by SOAs (9 levels, from −200 to +200 ms) was conducted. There was a significant main effect of SOA on the size of the distance effect (*F*(8, 384) = 3.91, *p* < .001, $\eta_{p}^{2}=.08$). The post hoc comparisons between mean RTs of adjacent SOA conditions showed that SOA conditions −200 ms and −150 ms, −50 ms and 0 ms, and 0 and +50 ms were significantly different from each other (all *ps* < .05), all other comparisons between mean RTs for adjacent SOA conditions were not significant. This result indicated a more negative beta value, that is, a larger distance effect, when the auditory and visual stimuli were displayed simultaneously, and then became larger (less negative, that is, a smaller distance effect) when the SOA increased. Post hoc analyses showed that the beta value of 0 ms SOA was significantly more negative than others (all *ps* < .05), indicating a significantly larger distance effect.

To further investigate the possible influence of modality order on the distance effect, a 2 (modality order: VA and AV) by 4 (SOA: 50, 100, 150, and 200 ms) ANOVA was conducted. A significant main effect was found for modality order (*F*(1, 48) = 5.17, *p* = .028, $\eta_{p}^{2}=.10$), indicating a larger distance effect in VA conditions (mean beta value = −.30, 95% confidence interval = [−.37, −.24]) than in AV conditions (mean beta values = −.21, 95% confidence interval = [−.27, −.16]). There was no main effect of SOA (*F*(3, 144) = 2.12, *p* = .10, $\eta_{p}^{2}=.04$). However, there was a significant linear trend of SOA (*F*(1, 48) = 5.15, *p* = .028, $\eta_{p}^{2}=.10$), indicating that beta values increased linearly with SOA. The interaction between modality order and the SOA was not significant (*F*(3, 144) = .54, *p* = .66, $\eta_{p}^{2}=.01$).

#### Model fitting

In the VA condition, the slope for $N_{D}$ (slope = −0.010, *t* = −6.77, *p* < .001), $P_{digit}$ (slope = −0.003, *t* = −2.64, *p* < .001), and the intercept (2.805, *t* = 396.98, *p* < .001) were significant (marginal *R*^2^ = .010; see Table 2). In the AV condition, the slope for $N_{D}$ (slope = −0.007, *t* = −4.19, *p* < .001) and the intercept (2.773, *t* = 375.44, *p* < .001) were significant (marginal *R*^2^ = .006). For simultaneous presentation, the slope for $N_{D}$ (slope = −0.021, *t* = −7.52, *p* < .001) and the intercept (2.835, *t* = 421.75, *p* < .001) were significant, while the slope for $P_{digit}$ (slope = −0.005, *t* = −2.32, *p* = .020) was marginally significant (marginal *R*^2^ = .048). Like in Experiment 1, we also examined different models at both ends of SOAs to see whether the results changed during this SOA interval, which were VA200 and AV200. In the VA200 condition, the slope for $P_{digit}$ (slope = −0.007, *t* = −3.23, *p* = .0013) and the intercept (2.791, *t* = 395.74, *p* < .001) were significant (marginal *R*^2^ = .009). At AV200 condition, the slope for $P_{auditory}$ (slope = 0.011, *t* = 3.70, *p* < .001) and the intercept (2.735, *t* = 332.16, *p* < .001) were significant (marginal *R*^2^ = .009). Only significant predictors were reported (Bonferroni corrected as in Experiment 1).

#### Relationship to mathematical performance

In contrast to experiment 1, there was no significant correlation (*r*(49) = −.04, *p* = .77)^6^ between WRAT standardised scores (*M* = 104.76, *SD* = 11.45, range from 87 to 137) and the averaged RT across SOA conditions for audiovisual matching task (see Figure 4(b)). The WRAT scores were also not correlated with the size of participants’ distance effect overall (overall beta values, *r*(49) = −.13, *p* = .36) or with the distance effect for simultaneous presentation only (beta values at 0 SOA, *r*(49) = .17, *p* = .25).

## General Discussion

To our knowledge, this study is the first to investigate the correspondence between spoken number words and Arabic digits systematically with SOA manipulations. A significant distance effect was found in both experiments and the distance effect was modulated by SOA. The distance effect was strongest for simultaneous cross-modal presentation. Both numerical distance and physical similarity between the stimuli were significant predictors of RTs. However, their relative contribution was modulated by SOA with a stronger contribution of numerical distance for simultaneous presentation and physical similarity as the only significant predictor of RTs at the longest SOAs. Our study does not give a clear answer about the relationship between participants’ RTs in the audiovisual matching task and their mathematical performance. In Experiment 1, there was a significant negative relationship, but we failed to replicate this correlation in Experiment 2. Thus, overall, there was no consistent relationship.

### Cross-modal numerical distance effect

The most important finding in this study is the modulation of the cross-modal distance effect by SOA. A cross-format distance effect has previously been reported with Arabic digits and *written* number words, indicating that there is a common semantic representation of numbers, at least for these visual formats of numbers (e.g., Dehaene & Akhavein, 1995). Our study further extends this finding: a common semantic representation may exist between different modalities of numbers to spoken number words and the extent of the activation of the semantic representation depends on the timing between the presentation of the Arabic digit and the spoken number word.

There are two opposing proposals for cross-modal number judgements. The first point of view assumes that all numerical stimuli, no matter of their input format, are converted into an amodal magnitude representation (e.g., McCloskey, 1992). Only then the quantity information is compared. Thus, a distance effect is always expected. Moreover, as all internal numerical operations (e.g., magnitude comparisons) are conducted in the same abstract format of numbers, this model does not predict that the distance effect interacts with number formats. While this model can explain the numerical distance effect present for simultaneous presentation in our data, it cannot account for the absence of the distance effect at the longest SOAs.

An alternate conception proposes multiple magnitude representations for different numerical inputs (e.g., Campbell, 1994; Campbell & Clark, 1988; Campbell & Epp, 2004; Cohen et al., 2013; Noël & Seron, 1993). In this view, there is no abstract representation of all number formats, instead the existence of direct, asemantic connections between different numerical inputs is proposed. For example, a written Arabic digit “5” can possibly activate the representation of its phonological counterpart /five/ via a direct route without having to access quantity information. In the multiple representation model (Cohen et al., 2013), same–different judgements for different numerical inputs are then made based on physical similarity between the stimuli. For example, when judging whether an auditory number word /two/ is same or different from a visual Arabic digit “3,” the auditory number word /two/ may be transcoded into the Arabic digit format “2.” Hence, a same–different judgement can be made during this stage by merely comparing the visual appearance of numerals, without accessing the quantity information of these numerals, for example, the Arabic digit “2” is visually different from “3.” Consequently, RTs on different trials are predicted to be related to the physical similarity between the two stimuli.

Testing this proposal, Cohen et al. (2013; Experiment 3) conducted an audiovisual experiment: auditory number words were displayed 500 ms earlier than the visual Arabic digits in a matching task. Visual similarity between Arabic digits was the only significant predictor of RTs providing evidence that participants converted the auditory number word into an Arabic digit and then assessed the visual overlap between the two Arabic digits. In their experiment, numerical distance was not a significant predictor.

This is in contrast to our results. In Experiment 1, when spoken number words were presented 500 ms before the presentation of the visual digit, visual overlap between Arabic digits did not predict RTs. In contrast, such visual overlap predicted reaction times when visual digits were presented 500 ms before spoken number words. Moreover, in Experiment 2, for the longest SOA, when spoken number words were presented 200 ms before the presentation of the visual digit, auditory similarity between the stimuli was the only significant predictor of RTs.

It is worth mentioning here that there are at least three differences between our experimental design and the design used by Cohen et al. (2013). First, they used a blocked design, which means that the auditory number words were always displayed before the visual digits in their experiment while our study employed a mixed design. The blocked design might have encouraged participants more strongly to employ strategies to facilitate their response speed, such as transcoding the first-displayed auditory number word into digit format, and especially given that participants had enough time (500 ms) for the transcoding. In contrast, in our experiments, the VA and AV trials were intermixed. Second, while Cohen et al. (2013) included 7 in their stimulus set, we did not use 7 in our stimulus list because the number word “seven” has two syllables. This is in contrast to all other number words for single digits which are monosyllabic. Third, we fit our data with a linear distance model rather than a logarithmic model. These design differences may account for some of the differences between our results and those by Cohen and colleagues.

While physical similarity does predict reaction times in some of our SOA conditions, our findings nevertheless provide strong evidence for the influence of numerical distance in addition to physical similarity. Overall, numerical distance emerged as the strongest and most consistent predictor of RTs, in particular at simultaneous presentation. When we entered physical similarity (visual and auditory) and numerical distance as predictors, numerical distance was a significant predictor when numbers were presented simultaneously and in both AV and VA conditions. Furthermore, in our data, the distance effect was significant for simultaneous presentation in both experiments, but physical similarity did only significantly predict RTs for simultaneous presentation in Experiment 1 and not in Experiment 2. One might argue that by applying Bonferroni correction, we might have been overly cautious. Without Bonferroni correction, visual physical similarity also emerges as a significant predictor of RTs for simultaneous presentation in Experiment 2. However, the contribution (beta weights) of numerical distance as a predictor is generally larger than the contribution of physical similarity as a predictor (see Table 2). Therefore, while visual physical similarity might also influence reaction times for simultaneous presentation, numerical distance was the strongest predictor for simultaneous presentation in both experiments. This provides evidence that the distance effect in our results is mostly driven by numerical distance and argues against the idea that the distance effect observed in our data is only driven by physical similarity. Our results thus do not support a model which only assumes non-abstract representations for quantity information.

The third approach to explain number processing is a combination of the two proposals discussed above, for example, the triple-code model (Dehaene, 1992). In this model, there is an analogue magnitude representation accounting for magnitude comparison, and there are also separate, special codes for processing auditory number words and visual Arabic digits. More importantly, in this model, there are both direct and indirect routes between auditory and visual codes. On one hand, the direct, asemantic route is somewhat similar to the idea of aforementioned multiple representation model, that is, without a need to pass through the analogue magnitude representation. Thus, the quantity information is not always necessary for a same–different judgement. On the other hand, these two codes can also communicate indirectly via the analogue magnitude representation, where the semantic quantity information of numerals is processed, leading to a numerical distance effect.

We found clear evidence for a numerical distance effect in reaction times in both experiments. In addition, there was evidence that physical similarity between the stimuli was a significant predictor of RTs, in particular at longer SOAs. Thus, our results are in line with models which allow an intermediate magnitude representation for auditory and visual numerical inputs, such as the abstract modular model (McCloskey, 1992) and the triple-code model (Dehaene, 1992). One possibility is that the presentation of a number triggers the activation of its semantic representation and the transcoding process in parallel (i.e., visual digit processing activates its phonological representation, for example, Roelofs, 2006). The reaction time pattern in a cross-modal matching task might then depend upon which information from both numbers is available first.

The direct, asemantic route between spoken number words and Arabic digits might become more important when there is more time to process the first-displayed numeral. At longer SOAs, participants may transcode the visual digit into the auditory number word format in the VA conditions, while they may transcode the auditory number word into a visual Arabic digit format in the AV conditions. Hence, when there is more time for transcoding of the first stimulus (longer SOA), the transcoding process is closer to completion, thus a same–different judgement becomes easier to make based on physical characteristics, without accessing the abstract magnitude representation. In contrast, when the SOA is close to zero, there is not sufficient time for participants to transcode the numeral into another format, thus they rely more on semantic quantity information for making responses.

It is worth mentioning that Sasanguie and Reynvoet (2014) also suggested the use of an asemantic route for audiovisual same–different judgements between Arabic digits and spoken number words. However, although they found longer reaction times for close-distance pairs than for far-distance pairs, the distance effect for simultaneous presentation was not significant in their study. The absence of the distance effect in their study could be due to their experimental design: for instance, only four number stimuli (number 1, 2, 8, and 9) were used. Participants might be able to establish the stimulus–response linkage when fewer possible pairings of audiovisual stimuli are displayed repetitively. Furthermore, there might be a trend of a distance effect: the RT of close-distance trials were 10 ms slower than far-distance trials in Sasanguie and Reynvoet’s study, which possibly indicates that the semantic numerical magnitude was still accessed, but was weakened by the experimental design.

However, while our current data cannot be explained without proposing the contribution of both a semantic and an asemantic route to cross-modal number matching, there are some puzzling results. Based on the transcoding explanation discussed above, for long SOAs, we would predict visual similarity between the two stimuli to be a significant predictor of RTs in the AV condition and auditory similarity in the VA condition. However, the pattern in our data is reversed: visual similarity is a significant predictor in both experiments for VA overall and also in the VA condition with the longest SOA in Experiment 2 and auditory similarity is a significant predictor in the AV condition with the longest SOA in Experiment 2. These results could be due to our mixed design which discourages stable transcoding strategies because the order of the modalities and the SOAs change from trial to trial.

Alternatively, it is possible that they are masking individual differences. Noël and Seron (1993), in their preferred-entry model of number processing, suggest that participants have a preferred numerical code and that any numerical input is initially transcoded into their preferred code. Thus, this model predicts individual differences in the asemantic route in a cross-modal number task. Participants with the preferred code of Arabic digits will transcode the spoken number word into an Arabic digit and participants with the preferred code of spoken number word will transcode the Arabic digit into a spoken number word, irrespective of their presentation order in the experiment. Future experiments will have to provide more conclusive evidence about the workings of the asemantic route in particular for cross-modal same–different tasks with long SOAs.

### Is the performance in the audiovisual matching task related to participants’ arithmetic performance?

In contrast to the robust distance effect observed in Experiments 1 and 2, the relationship between participants’ RT of audiovisual matching task and their mathematical performance was inconsistent. A significant negative correlation was found in Experiment 1, indicating that participants who responded faster in the audiovisual matching task performed better in the mathematical standardised test. However, no significant correlation was found between participants’ mean RT of digit–number word matching task and their mathematical performance in Experiment 2. This may indicate the correlation is inconsistent across different participant groups or/and a small effect size.

Sasanguie and Reynvoet (2014) reported a correlation between the RT of an audiovisual matching task and mathematical performance (*r*(48) = −.36, *p* = .01^7^) of similar size to our first experiment. In addition to a digit–number word matching task, they also gave participant two additional matching tasks (e.g., dot–number word matching and letter–speech sound matching tasks) and control tasks (e.g., Raven IQ test and a general processing speed task). A hierarchical regression analysis showed that the digit–number word matching task (the same audiovisual matching paradigm in this study) significantly contributed to the variance of mathematical performance on top of the two other matching tasks and control tests. Lyons, Price, Vaessen, Blomert, and Ansari (2014) also reported a significant correlation between reaction times of a computerised audiovisual matching task and arithmetic performance in a large sample of children (*N* > 1,000, from Grades 1-6). However, in a backward step-wise regression predicting arithmetic, the audiovisual matching ability was removed as a predictor in the final model when age, IQ, reading, counting ability, and seven other numerical skills were entered. In a more recent paper with 60 adults, Sasanguie, Lyons, De Smedt, and Reynvoet (2017) failed to find a significant relationship between performance on the matching task between digits and spoken number words and arithmetic performance. Thus, so far, the evidence that adults who are faster in comparing numerical stimuli across modalities are those who also show higher performance on tests of mathematical performance is mixed at best.

## Conclusion

To conclude, this study highlights two main findings: First, both experiments provide evidence for a cross-modal numerical distance effect. Second, the largest distance effect was found when the auditory and visual stimuli were presented simultaneously, and the distance effect became smaller the longer the time between the onset of the visual and the auditory number. These results clearly suggest that the magnitude representation is accessed for numerical judgement when the audiovisual numerals are given in close temporal proximity to each other. Our results also hint at the possible involvement of an asemantic route between spoken number words and Arabic digit that gets stronger when Arabic digits and number words are presented with larger temporal asynchrony.

## Notes

1. All the post hoc analyses of analysis of variance (ANOVA) in this article were Bonferroni corrected if not mentioned otherwise.

2. Post hoc analyses showed that response time (RT) of auditory first-then-visual (AV) conditions significantly decreased when the stimulus onset asynchronies (SOAs) increased (all *p* values of the SOA next to itself ⩽.001). In contrast, in visual first-then-auditory (VA) conditions, only the mean RT of VA 100 ms was significantly longer than VA 200 ms (*p* < .001). The mean RT for VA 200 ms was not significantly different from VA 300 ms (*p* > .45) or VA 500 ms (*p* > .77).

3. We used a Bonferroni correction. As there were four predictors, the significant criterion for *p* values was .05/3 = .0166.

4. The interaction was due to the different patterns between same and different trials from −200 to −100 ms SOA conditions. The slope for the same trials from −200 to −150 ms was steeper than from −150 to −100 ms, whereas the RT pattern for different trials here was reversed, flatter from −200 to −150 ms but steeper from −150 to −100 ms. These different RT patterns within the matching factor were not further addressed because this study focused on the RT of numerically different trials.

5. More specifically, post hoc analyses showed that RTs of AV conditions significantly decreased when the SOA increased in both same and different trials (all *p* values of adjacent SOA conditions ⩽.01). In contrast, in same trials, RTs of VA 100 ms (*M* = 624 ms, *SD* = 80 ms) were significantly longer than VA 200 ms (*M* = 624 ms, *SD* = 80 ms; *p* = .018), but VA 200 ms was not significantly different from VA 300 ms (*M* = 624 ms, *SD* = 80 ms; *p* > .99) or VA 500 ms (*M* = 624 ms, *SD* = 80 ms; *p* = .22); in different trials, RTs of VA 100 ms were not significantly different from VA 200 ms (*p* = .17) and RTs of VA 300 ms were not significantly different from VA 500 ms (*p* > .99).

6. Because the Pearson correlation was not significant, partial correlations controlling the contribution of non-verbal IQ (mean raw scores = 22.5, *SD* = 2.7; mean *T*-scores = 55.12, *SD* = 8.0) and general processing speed (*M* = 270 ms, *SD* = 29 ms) are not reported.

7. This was a partial correlation in which individual IQ and processing speed were controlled.
